# The Potential Nexus between Helminths and SARS-CoV-2 Infection: A Literature Review

**DOI:** 10.1155/2023/5544819

**Published:** 2023-06-20

**Authors:** Hayder M. Al-kuraishy, Ali I. Al-Gareeb, Luay Alkazmi, Maisra M. El-Bouseary, Rabab S. Hamad, Mahmoud Abdelhamid, Gaber El-Saber Batiha

**Affiliations:** ^1^Department of Clinical Pharmacology and Medicine, College of Medicine, Al-Mustansiriya University, Baghdad, Iraq; ^2^Biology Department, Faculty of Applied Sciences, Umm Al-Qura University, Makkah 21955, Saudi Arabia; ^3^Department of Pharmaceutical Microbiology, Faculty of Pharmacy, Tanta University, Tanta, Egypt; ^4^Biological Sciences Department, College of Science, King Faisal University, Al Ahsa 31982, Saudi Arabia; ^5^Central Laboratory, Theodor Bilharz Research Institute, Giza 12411, Egypt; ^6^Department of Parasitology, Faculty of Veterinary Medicine, Aswan University, Aswan 81528, Egypt; ^7^Department of Pharmacology and Therapeutics, Faculty of Veterinary Medicine, Damanhour University, Damanhour 22511, AlBeheira, Egypt

## Abstract

Chronic helminth infections (CHIs) can induce immunological tolerance through the upregulation of regulatory T cells. In coronavirus disease 2019 (COVID-19), abnormal adaptive immune response and exaggerated immune response may cause immune-mediated tissue damage. Severe acute respiratory syndrome-coronavirus 2 (SARS-CoV-2) and CHIs establish complicated immune interactions due to SARS-CoV-2-induced immunological stimulation and CHIs-induced immunological tolerance. However, COVID-19 severity in patients with CHIs is mild, as immune-suppressive anti-inflammatory cytokines counterbalance the risk of cytokine storm. Since CHIs have immunomodulatory effects, therefore, this narrative review aimed to clarify how CHIs modulate the immunoinflammatory response in SARS-CoV-2 infection. CHIs, through helminth-derived molecules, may suppress SARS-CoV-2 entry and associated hyperinflammation through attenuation of the inflammatory signaling pathway. In addition, CHIs may reduce the COVID-19 severity by reducing the SARS-CoV-2 entry points in the initial phase and immunomodulation in the late phase of the disease by suppressing the release of pro-inflammatory cytokines. In conclusion, CHIs may reduce the severity of SARS-CoV-2 infection by reducing hyperinflammation and exaggerated immune response. Thus, retrospective and prospective studies are recommended in this regard.

## 1. Introduction

Helminths can be divided into trematodes, cestodes, or nematodes, an evolutionarily diverse group of organisms. Parasitic diseases represent a major human health concern, a significant impact on livestock productivity, and a common problem in companion animals [1, 2]. Helminths are important metazoan parasites that affect about one-third of the world's population, evading and regulating the host immune response [1]. Associated with the resting of human immune reactivity through regulation of the host immune response, helminths are regarded as human body's xenotransplants, whose interaction with the host immune system leads to damping of the host immune response to autoantigens and allergens by modulating the host immune response to specific antigens [3–5].

Infection with a helminth parasite is a potent stimulus of host immunity that seeks to destroy/inactivate and eradicate the worm, while the parasite strives to circumvent the host's antiworm efforts, allowing establishment, access to nutrients, and completion of its' life cycle [6]. Epidemiologically, with the exception of *Strongyloides*, helminths cannot multiply or reproduce within the human host but can survive for many years in tissues and intestinal niches. This long stay of helminths has been related to immunological tolerance, hyporesponsiveness, and modulation of immune response [7]. Therefore, clearance of helminths through the use of anthelminthic drugs in immune-tolerant carriers may trigger specific antigen responses and enflame immune stimulation, suggesting the hindering role of helminths in humans' immune responses [8]. Helminths have systemic immunomodulatory effects triggered by their excretory/secretory helminth (E/S) products and may influence the severity of other infections [9]. These helminth products (E/S) induce the host Th2 immune responsiveness mediated by regulatory T cells (Treg), which can expulse the helminth parasite [10].

Likewise, T cells from asymptomatic subjects with helminthic infection illustrate the favorable anti-inflammatory cytokine profile, while in symptomatic patients, the immunological tolerance is reduced, leading to tissue damage due to exaggeration of the immune response to helminth antigens [11]. Indeed, it has been reported that the immunological tolerance to harmless and self-antigens is mediated by Treg, which are upregulated in chronic helminth infections (CHIs) [12]. Briefly, the Treg cells in helminth-infected patients express a forkhead box protein 3 (Foxp3), which reduce the anthelmintics activity since Foxp3 serum level is correlated with Treg cells level and decreased allergic response [13]. For this reason, the immunological response to vaccines is attenuated in children infected with helminths. As well, an exaggerated human immune system response against malaria and pulmonary tuberculosis is reduced in CHIs patients [14]. Therefore, helminth infection attenuates immune-mediated tissue injury by modulating immune and inflammatory responses, mostly by upregulating anti-inflammatory interleukin (IL)-10 levels [14, 15].

**Figure 1:**
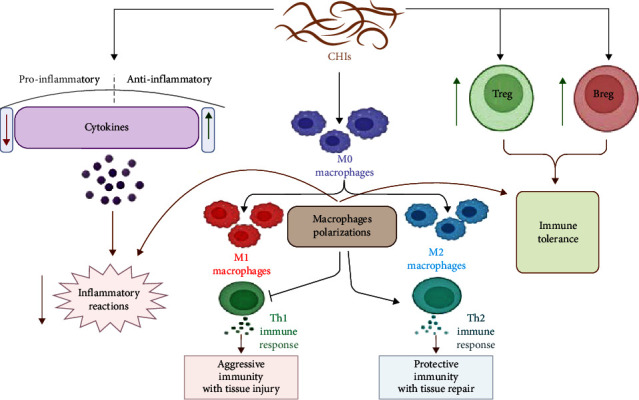
Chronic helminth infections (CHIs) and immunomodulation: CHIs increase anti-inflammatory cytokines and inhibit pro-inflammatory cytokines, leading to a reduced progression of inflammatory reactions. Besides, CHIs increase the development of regulatory B cells (Breg) and regulatory T cells (Treg), leading to the development of immune tolerance. In addition, CHIs trigger polarization of macrophages from classical (M1) to alternative macrophages (M2) that activate type 2 immune response (Th2) and inhibit type 1 immune response (Th1), leading to improvement of protective immunity.

It has been proposed that CHIs-mediated immune-modulating effects involve: (1) modulation of cytokine production toward anti-inflammatory axis with reduction of pro-inflammatory cytokines [16]; (2) downregulation of cell-surface interactions through activation of cytotoxic T cells and programed cell death [7]; (3) driving of human immunoglobulin G (IgG) to be noninflammatory IgG4 by Treg dependent-Foxp3 production [17]; (4) activation of host cells to produce tumor growth factor beta (TGF*β*), which provokes Treg cell function; (5) upregulation of regulatory B cells (Breg), triggering immunological tolerance to helminth infections [18]; (6) alteration in macrophage response from classically activated macrophage (M1) to alternatively ones (M2), where helminth-stimulated M2 leads to activation of Treg and induction of immune tolerance [19] (Figure 1).

The interaction between helminths and the host's immune system triggers specific immunomodulatory and immunoregulatory mechanisms that guarantee their survival in the host for years [20]. Nevertheless, these changes might weaken the immunological response to bystander bacterial, viral, and protozoal pathogens [20]. Modulation of the immune system by infection with helminthic parasites is suggested to reduce the levels of allergic responses and to protect against inflammatory diseases [20, 21]. Helminth-induced immunoregulatory mechanisms that suppress protective responses to human immune deficiency virus (HIV) could be detrimental [22]. Although the function, phenotype, and antigen-specificity of CD4/CD25 Treg have been implicated in the modulation of immune responses to bystander antigens and could suppress HIV-specific CD4- and CD8-derived cytokine production and lymphocyte proliferation, suggesting that they may play a role in suppressing antiviral immune responses [23]. On the other hand, regulatory activity could have benefits: replication of proviral DNA depends on the activation of host cell transcription factors, so helminth-induced regulatory activity that suppresses such transcription could be beneficial, particularly in relation to HIV progression [22, 23]. Despite these findings, several studies have demonstrated a negative association between helminth infections and inflammatory diseases. Other found point in the opposite direction. The discrepancy may be explained by differences in frequency, dose, time, and type of helminth [24, 25]. These findings suggest a bidirectional effect of CHIs on viral infection that might be beneficial or detrimental.

Coronavirus disease 2019 (COVID-19) is a worldwide pandemic disease caused by the severe acute respiratory syndrome-coronavirus type 2 (SARS-CoV-2) [26]. COVID-19 is associated with an exaggerated immune response and the development of hyperinflammation [27]. Since CHIs have immunomodulatory effects [16]; therefore, this narrative review aimed to clarify how CHIs modulate the immunoinflammatory response in SARS-CoV-2 infection.

## 2. Immune Response for SARS-CoV-2 Infection

SARS-CoV-2 spike protein binds to the angiotensin-converting enzyme 2 (ACE2), which acts as a receptor for the SARS-CoV-2 entry inside cells [28–30]. ACE2 is highly expressed in different tissues, including lung pneumocytes, enterocytes, endothelial cells, and cardiomyocytes [29], and the interaction between SARS-CoV-2 and ACE2 results in the downregulation of the protective ACE2 with the initiation of hyper-inflammation and oxidative stress, that cause acute lung injury (ALI), acute respiratory distress syndrome (ARDS), and multiorgan failure [30–32].

**Figure 2:**
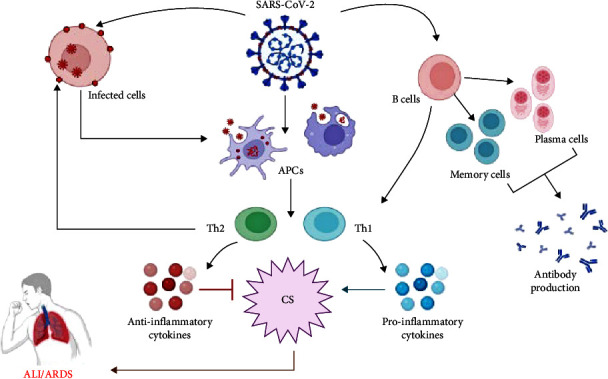
Immunological interaction in SARS-CoV-2 infection: SARS-CoV-2 activates antigen-presenting cells (APCs), which triggers the activation of T cells with the development of type 1 immune response (Th1) and type 2 immune response (Th2). Th1 activates releases of pro-inflammatory cytokines with the development of cytokine storm (CS), while Th2 activates releases of anti-inflammatory cytokines, which inhibit CS and the development of acute lung injury (ALI) and acute respiratory distress syndrome (ARDS).

Besides, the SARS-CoV-2 infection can simultaneously provoke both adaptive and innate host immunity [33]. However, an impaired adaptive immune response and an unrestrained innate immune response could result in extensive local and systemic immune-mediated tissue damage [34]. Qin et al. [35] found that patients with severe COVID-19 presented with lymphopenia and a significant reduction in the number of natural killer (NK), B cells, monocytes, basophils, eosinophils, CD4 and CD8 T cells. In addition, the neutrophil–lymphocyte ratio (NLR) was increased, which directly correlated with COVID-19 severity and the development of ALI and ARDS [35] (Figure 2).

The humoral immune response in SARS-CoV-2 infection is reflected by elevations of IgM and IgG that are correlated with clinical improvement in COVID-19 patients [36, 37]. Conversely, two prospective studies conducted by Zhao et al. [38] and Zhang et al. [39] found that COVID-19 severity is linked to exaggerated IgG levels, leading to poor clinical outcomes due to the antibody-dependent enhancement effects (ADE) for SARS-CoV-2 entry into the host cells and induction of pro-inflammatory reactions. The ADE effect was stated in the pathogenesis of the Middle East respiratory syndrome in 2012 [39]. Therefore, high IgG levels, besides its antiviral activity, may lead to secondary organ damage through the recruitment of monocytes, macrophages, and the production of pro-inflammatory cytokines [38]. Furthermore, high pro-inflammatory cytokine levels, including IL-2, IL-6, TNF-*α*, IL-17, and IL-18, can lead to cytokine storm (CS) induced-diffuse organ damage, shock, ALI, and ARDS [40]. Consequently, modulation of exaggerated immune responses and pro-inflammatory cytokines by IL-6 receptor monoclonal antibody (tocilizumab), complement activation inhibitors, anti-inflammatory, and immunosuppressive agents may reduce the likelihood of ALI in COVID-19 patients [41, 42].

In general, COVID-19 is asymptomatic and produces mild illness in about 85% of infected individuals. However, about 10% of infected individuals lead to moderate-to-severe pulmonary and extra-pulmonary manifestations characterized by fever, headache, anosmia, myalgia, diarrhea, and thrombotic events. Overall, 5% of infected subjects may develop a critical illness due to the development of CS, ALI, and ARDS that required intensive care admission and mechanical ventilation [43–48]. It has been shown that COVID-19 severity is linked with exaggeration of immune response, hyperinflammation, and hypercytokinemia [49].

These observations indicated that SARS-CoV-2 infection in severe conditions may lead to exaggeration of immune response with the development of critical complications like CS.

## 3. Effects of Helminths on the COVID-19 Outcomes

Helminths immune modulation has the ability to suppress inflammatory responses present during infection by protozoon, bacteria, and virus [50]. The immunological interactions between SARS-CoV-2 infection and CHIs are complicated and not simply defined by SARS-CoV-2-induced immunological stimulation and associated helminth immunological changes. CHIs may have beneficial and detrimental effects on COVID-19.

### 3.1. Beneficial Effects

Of note, helminth infections can decrease the risk of metabolic disorders like metabolic syndrome, type 2 diabetes mellitus (T2DM), and obesity that are commonly associated with higher COVID-19 severity [51]. Definitely, lymphopenia and CS are more common among COVID-19 patients with underlying metabolic disorders. As well, eosinopenia and reduction of Treg are closely linked to COVID-19 severity [52]. Therefore, CHIs may reduce the severity of the potential risk for COVID-19 severity. Different epidemiological studies have confirmed that CHIs reduce the risk of T2DM and insulin resistance (IR) through the modulation of pro-inflammatory cytokines [53]. Various studies have shown an inverse connection between CHIs and inflammatory diseases such as allergies, autoimmunity, and inflammatory bowel disease, but importantly there is emerging evidence that helminths seem to also be associated with a lower incidence of metabolic syndrome [54].

It has been proposed that CHIs are associated with elevations of Treg cells, eosinophilia, and M2 macrophages while reducing the capacity for the production of pro-inflammatory cytokines [55]. High Treg cells and eosinophilia are associated with a low rate of CS and good clinical outcomes in COVID-19 patients [56, 57].

CHIs have been illustrated to have protective effects against the development of ALI in viral pneumonia via the upregulation of anti-inflammatory cytokines (IL-4, IL-10) [58]. In addition, enteric helminthiasis has remote antiviral effects against viral pneumonia through modulation of microbiota-dependent immune response, high diversity of intestinal microbiota, mainly *lactobacillaceae*, and was associated with a reduction in viral-induced lung damage through assembly of microbiota-dependent anti-inflammatory cytokines [56]. Furthermore, a prospective study involved 515 patients with positive polymerase chain reaction for the presence of SARS-CoV-2, screened them for CHIs, of whom 267 (51.8%) were coinfected with different helminths. The study revealed that COVID-19 severity was lower in coinfected patients (19.0%, CI: 14.52–24.35) due to the immune-modulatory effect of CHIs on the pro-inflammatory milieu in patients with severe COVID-19 [59].

Generally, in healthy subjects, both type 1 and type 2 immune responses are involved in homeostasis, helping to prevent dysregulation of immune response [60]. This host–helminth interaction could be beneficial in dampening inflammatory damage induced by the Th1/Th17 branches of the immune system, repairing injured tissue, and restoring homeostasis [60]. However, in COVID-19 and other viral infections, type 1 immune response is dominating, leading to hypercytokinemia with high pro-inflammatory cytokines levels, including IL-6, tumor necrosis factor (TNF)-*α*, and IL-8 in COVID-19 patients, and is linked to a higher risk of developing ARDS and poor clinical outcomes [61]. Different studies proposed that the immunosuppressive and Treg response stimulated by helminths may balance the inflammatory Th1/Th17 response triggered by SARS-CoV-2 infection, potentially restricting the severity of COVID-19 disease [52, 62]. Though the COVID-19 severity in patients with preexisting CHIs is mild, in most cases, due to the effect of immunosuppressive anti-inflammatory cytokines that counteract the risk of CS [63]. Downregulation of pro-inflammatory cytokines by parasites may reduce the probability of developing CS, as observed in patients with COVID-19 or with other viral infections [64]. Therefore, regular deworming in such individuals should be practiced due to mild-to-moderate protection against COVID-19 complications [65]. For example, the low-frequency rates of COVID-19 in Africa are an extraordinary concern for scientists, and it has been theorized that this could be an effect of the augmented exposure to parasites in less developed countries (e.g., African and Latin American populations are much more likely to suffer from parasitic diseases than those living in more developed countries) [66].

It has been shown that COVID-19 lethality rates are significantly lower in sub-Saharan Africa than in the industrialized world [67]. Coinfection with the enteric parasites, *Hymenolopis nana*, *Schistosoma mansoni*, and *Trichuris trichiura* reduced the risk of severe COVID-19 occurrence in this cohort of African patients. When stratified by species, coinfection with *T*. *trichiura* showed the lowest probability of developing severe COVID-19 [68]. These observations proposed that CHIs induced a Th2-prone response in the host, which modulates COVID-19 severity by restricting the hyper-inflammation associated with the viral infection. The prevalence and severity of SARS-CoV-2 infection and associated pneumonia are higher in developed societies where helminth infection is uncommon [69]. These outcomes suggest a protective role of CHIs against COVID-19 severity.

### 3.2. Detrimental Effects

In contrast, CHIs with viral coinfection may promote virus-associated lung disease by downregulating viral-specific immune response [70]. Development of Treg and IL-10 production induced by CHIs impair the host immune responses, which in turn increases host susceptibility to microbial infections [71]. An experimental study on parasitic worm and virus coinfection in mice demonstrated that the immunomodulatory role of helminths creates a fortunate environment for the worms at the expense of antiviral immunity [72]. The suppression of the antiviral response by CHIs-induced immunomodulatory effects may encourage viral replication and result in a delay of clearance of SARS-CoV-2 [70, 73]. A prolonged exposure to parasitic helminth infection has been associated with generalized immune hyporesponsiveness, some of those cytokines, mainly TGF*β*, can trigger pulmonary fibrosis and fibrotic sequelae of pulmonary tuberculosis in helminth infection settings [74].

It has been suggested that the increased severity and mortality of COVID-19 in helminth endemic areas may be attributed to the nutritional and metabolic compromises caused by worm infections [75]. Thus, deworming in COVID-19 patients as it may decrease SARS-CoV-2 viral load and improve CD8 T cells in the lung microenvironment [76]. Notably, COVID-19 patients coinfected with helminths may be unable to support a rapid and effectual immune response against SARS-CoV-2 in the early phase of the infection, thus leading to increased patient morbidity and mortality [77]. CHIs attenuate host immune response, thereby reducing vaccine efficacy and augment COVID-19 severity, which may increase the morbidity and mortality [77]. Therefore, treatment and prevention of helminth infections in endemic regions might decrease the morbidity and mortality of COVID-19. CHIs-induced malnutrition and anemia may increase the severity of COVID-19 [78]. However, Long et al. [79] showed that an initial weak immune response in patients with COVID-19 leads to a lower risk of developing CS and ARDS. Indeed, the main cause of COVID-19 severity is exaggerated immune responses that are linked to the progression of ALI and ARDS [33]. Therefore, immunosuppressive agents may be effective in the late phase of COVID-19 by dampening the CS. However, these agents may reduce the initial immune response that is required to control viral replication [80]. Nonetheless, both cyclosporine and tacrolimus are effective against viral replication in the early phase and prevent CS in the late stage of infection [81, 82].

The collapse of innate immunity, especially the presence of exhausted NK cells in severe cases of COVID-19 associated with CS, is considered an important mechanism that is associated with disease severity and critical illness [83]. NK cells in severe COVID-19 patients may provide an effective cure, especially in cases suffering from CS [83]. Therefore, NK cells to halt CS, it is very likely that this strategy will be effective in preventing either acute conditions of COVID-19 or in the treatment of severe forms of this disease. Besides, dysfunctional innate immunity may aggravate CHIs status [84]. Gentile et al. [84] observed that NK-cell recruitment limits tissue damage during an enteric helminth infection. The depletion of tissue-infiltrating NK cells altered worm burden and increased vascular injury, suggesting a role for NK cells in mediating tissue protection. Together, these data identify an unexpected role for NK cells in promoting disease tolerance during the invasive stage of an enteric helminth infection [84]. Therefore, the depletion of NK cells in severe COVID-19 may aggravate tissue injury in patients with underlying CHIs.

Nevertheless, the shortage and lack of large-scale epidemiological studies regarding COVID-19 prevalence, mainly in developing countries, can be viewed as an obstacle to finding the association between COVID-19 severity and CHIs [73]. Thus, these verdicts indicated that CHIs may increase COVID-19 severity by inhibiting of innate/adaptive immune response.

## 4. Helminth Infections and SARS-CoV-2 Infection

### 4.1. Immune Response

One of the major pathways for nuclear factor kappa-B (NF-*κ*B) activation after COVID-19 infection is the MyD88 pathway through pattern recognition receptors (PRRs), leading to the induction of a variety of pro-inflammatory cytokines initiate CS, including IL-6, TNF-*α*, and chemokines [85]. PRRs play crucial roles in the innate immune response by recognizing pathogen-associated molecular patterns (PAMPs) and molecules derived from damaged cells, referred to as damage-associated molecular patterns [86]. Helminth parasites are masters at manipulating host immune responses using an array of sophisticated mechanisms. Early recognition of helminths by the host pulmonary innate immune response is critical for disease control. One of the major mechanisms enabling helminths to establish chronic infections is the targeting of PRRs [87]. In fact, it has been proposed that helminth molecules have the ability to block the effect of PRRs as part of their immunomodulatory effect [88]. Moreover, helminth products shares important features in that the products generally fail to suppress activation by pro-inflammatory PAMP, which results in the impairment of Th1 development and a bias of the immune response toward Th2 or Treg [89]. Thus, CHIs can alleviate the severity of COVID-19 by decreasing the CS progression. Nonetheless, some helminth products have also been shown to prime Th2 or regulatory responses through the inhibition of Toll-like receptors (TLR). For instance, excretory/secretory-62, a phosphorylcholine-containing glycoprotein of the nematode *Acanthocheilonema viteae*, induces Th2 responses through modulation of TLR4 [88]. TLR4 plays an important role in the recognition of viral particles and activation of the innate immune system. Activation of TLR4 pathways leads to the secretion of pro-inflammatory cytokines in COVID-19 infection. Thus, TLRs could be a potential target in controlling the infection in the early stages of the disease and production of vaccine against SARS-CoV-2 [90]. Therefore, modulation of PRRs/TLR4 by CHIs may reduce exaggeration immune response seen in patients with severe COVID-19.

### 4.2. SARS-CoV-2 Entry

CHIs can reduce the SARS-CoV-2 entry by reducing the expression of ACE2 receptors through activation of type 2 immune response [62]. Some helminths produce a local pulmonary anti-inflammatory effect involving a reduction in the expression of pulmonary ACE2, thereby reducing the viral load and inflammation-induced ALI [62]. As a result, CHIs stimulate immunosuppressants and the Treg branch of the immune system, which reduces the expression of ACE2 receptors that balance the inflammatory Th1 branch of the immune system, stimulated by SARS-CoV-2 [62]. However, ACE2 expression in allergic airway disease may decrease the risk and severity of COVID-19 [91]. Actually, CHIs have no direct effect on the expression of ACE2 but induced Th2 immune response by CHIs reduced ACE2 expression. For example, allergic asthma, which is triggered by the Th2 immune response, has been linked to a reduction in ACE2 expression; allergic asthma may offer some protection from severe COVID-19 [92]. Moreover, the dysregulated ACE2 expression in COVID-19 patients may lead to a high level of circulating AngII, causing pulmonary vasoconstriction and ALI [93]. Therefore, CHIs may attenuate ALI/ARDS in SARS-CoV-2-infected patients through the regulation of ACE2/AngII.

Of note, CHIs provoke the expression and release of anti-inflammatory cytokine IL-10, which reduces immune response and IgE signaling molecules [94]. IL-10 serum level is increased in severely affected COVID-19 patients as a compensatory mechanism to mitigate exaggerated immune response [95]. Freitas et al. [96] exhibited that IL-10 reduces vascular dysfunction by increasing the pulmoprotective effect of Ang (1–7). Therefore, CHIs may abrogate SARS-CoV-2-induced ALI via the IL-10/Ang (1–7) axis in COVID-19 pneumonia. Indeed, the high levels of Treg cells in CHIs patients attenuate AngII-induced ALI and ARDS [97].

Furthermore, dipeptidyl peptidase-4 (DPP4) plays an important role in SARS-CoV-2 entry, and thus, DPP4 inhibitors (DPP4Is) may hinder both SARS-CoV-2 entry and pathogenesis [98]. High expression of DPP4 receptors in immune cells and adipose tissue has been associated with obesity, T2DM, hypertension, metabolic syndrome, and chronic inflammatory disorders [99]. A recent experimental study stated that the transmembrane protease serine protease-2 (TRMPSS2) is increased in obese mice, facilitating the SARS-CoV-2 trimming and entry through both ACE2 and DPP4 receptors [100]. Different recent studies have shown the protective effect of DPP4Is in the management of COVID-19 patients, even in non-diabetic ones [101]. Notably, IL-10 can reduce the expression of DPP4 [102]; therefore, CHIs, through induction of IL-10, may reduce the expression of DPP4 receptors. Thus, chronic CHIs, through their anti-inflammatory effect, attenuation of IR, T2DM, and obesity, may reduce the expression of harmful DPP4 receptors and TRMPSS2, thereby constraining the SARS-CoV-2 binding to harmful receptors.

As well, CD147 is suggested as an important target for SARS-CoV-2 entry, and thus, inhibition of these receptors by azithromycin may be valuable in COVID-19 management [103]. In an *in silico* approach, Haçarız and Sayers [104] found a strong similarity between transcripts of immunomodulation-related *Fasciola hepatica* protein and CD147. Therefore, chronic *F. hepatica* infection may attenuate the SARS-CoV-2 infection by binding to immunomodulation-related protein instead of CD147 due to the strong similarity in the protein structure between CD147 and transcripts of *F. hepatica*. Therefore, *F. hepatica* acts as a modulator of SARS-CoV-2 infection and of COVID-19 pathology [105].

These observations indicated that CHIs could reduce SARS-CoV-2 entry through modulation expression of ACE2, DPP4, and CD147.

### 4.3. Activation of Nod-Like Receptor Pyrin Domain 3 (NLRP3)-Inflammasomes

CHIs may aggravate COVID-19 severity through the activation of inflammasomes, which is a multiprotein complex that controls the activation of pro-inflammatory cytokines through intracellular caspase-1 [105]. The NLRP3 is the most common inflammasomes engaged in immunity against different pathogens. NLRP3-inflammasomes activate type 1 immune response and inhibit type 2 immune response in CHIs [106]. It has been reported that both *S. mansoni* and *Heligmosomoides polygyrus* activate NLRP3-inflammasomes leading to the induction of pro-inflammatory cytokines [107, 108]. However, *F. hepatica* molecules have an important inhibitory effect on NLRP3 activation, in addition to preventing the development of type 1 immune response [109]. Freeman and Swartz [110] showed that SARS-CoV-2 infection is linked to NLRP3 activation, which is inhibited by double corticosteroid-interferon administration, which improves clinical outcomes in patients with severe COVID-19. Also, colchicine attenuates the SARS-CoV-2 infection through the inhibition of NLRP3 [80, 111].

Use of corticosteroids and immunomodulatory agents in COVID-19 patients may increase the risk for *Strongyloides* infection [112]. Strongyloidiasis is often asymptomatic in immunocompetent adults but may present with mild gastrointestinal or respiratory symptoms or with larva currens, a rapidly moving pruritic linear skin eruption. COVID-19 patients with undiagnosed *Strongyloides* infection undergoing immunosuppression are at risk of developing *Strongyloides* hyperinfection syndrome [112]. In addition, extensive use of corticosteroids for COVID-19 treatment has led to *Strongyloides* reactivation and severe disease in patients from endemic areas [113]. Activation of NLRP3-inflammasomes is more common in *Strongyloides* infection [15]; therefore, this type of helminths aggravate COVID-19 pathology and hyperinflammation. However, the interaction between COVID-19 and CHIs at the level of NLRP3-inflammasomes needs to be verified by large-scale experimental and clinical studies.

### 4.4. Eosinopenia in SARS-CoV-2 Infection

SARS-CoV-2 infection excites a multifaceted activation of the immune system. Eosinophils belong to the host's defense equipment against respiratory viruses. In the early phase of the infection, eosinophils contribution is probably appropriate and beneficial, as they facilitate the suppression of the viral replication. However, in severe COVID-19 patients, during the second and third phases of the disease, eosinophils may participate in a maladaptive immune response and directly contribute to immunopathology [114]. It has been verified that both viral load and eosinopenia in SARS-CoV-2 infection are linked to COVID-19 severity [115]. An observational and prospective study illustrated that eosinopenia <10/*μ*L is a useful, low-cost, reproducible tool to help diagnose COVID-19, during an epidemic period, in a population of hospitalized patients admitted for suspicion of COVID-19 [116]. A multicenter and retrospective study revealed that eosinopenia correlated with COVID-19 severity and was not a good predictor of mortality [117]. Certainly, despite the main cause of eosinopenia in COVID-19 is unclear, it is most likely due to immune-mediated reduction of the eosinophil life cycle through their migration and adhesion, and induction of apoptosis through the action of high INF-*γ* levels during acute infections [115]. Eosinophils' expansion and activation are stimulated by TH2 cytokines, especially IL-5, that correlated with SARS-CoV-2 clearance in COVID-19 [118]. Eosinophilia is a common finding in tropical developing countries and is mainly caused by CHIs, predominantly of the gut. Although only a minority of infections are symptomatic, development during childhood can be impaired, and in some patients, serious complications and sequelae may occur [119]. Eosinophilia in helminth infection is typically associated with a strong Th2 immune response, and eosinophils can effectively kill or damage larvae and adult worms in vitro. Eosinophils are involved in a range of immunomodulatory effects, such as increased production of the down-modulatory cytokines IL-10 and TGF*β*, as well as stimulation of Treg cells and alternatively activated macrophages [119]. These immunoinflammatory may reduce hyperinflammation in SARS-CoV-2 infection and other viral infections. Therefore, IL-5-dependent eosinophilia in CHIs can reduce COVID-19 severity through the antiviral and anti-inflammatory effects of high eosinophil counts [52].

### 4.5. Helminth and COVID-19 Severity

CHIs may increase the risk of secondary bacterial infections in COVID-19 patients, as well as attenuate the vaccine efficacy and inhibit the long-term immunity against SARS-CoV-2 due to suppression of the immune response against intracellular microorganisms causing diseases like tuberculosis, HIV, and opportunistic respiratory bacterial infections [63, 67, 120]. Therefore, CHIs and SARS-CoV-2 coinfection can lead to more critical outcomes, particularly in patients with underlying HIV and tuberculosis [121]. However, secondary bacterial infections are uncommon in COVID-19, unlike those that occur in influenza pneumonia, which has a high rate of severe secondary bacterial infections [122, 123]. In addition, CHIs can weaken the preliminary immune response during the initiation of SARS-CoV-2 infection, leading to a high viral load, decreased viral clearance, and prolongation of the infection length [77, 124].

CHIs are among the most common infectious diseases, despite the negative interactions between helminth infection and COVID-19 severity in helminth-endemic regions, and the alterations in the gut microbiome associated with helminth infection appear to have systemic immunomodulatory properties [125]. It has also been shown that helminth coinfection may increase COVID-19 morbidity and mortality, because the immune system cannot professionally respond to the virus [77, 126, 127]. As a result, vaccines will also be less effective for these patients, but treatment and prevention of helminth infections may reduce the negative effect of COVID-19 [63]. During times of host–parasite coevolution, helminths have evolved mechanisms to suppress the host immune responses, which may alleviate vaccine efficacy and increase the severity of other infectious diseases. Thus, helminth-viral coinfections can weaken immunity against SARS-CoV-2 and subsequently upsurge the hazard of COVID-19 [77].

Furthermore, a clinical trial aiming to assess the efficacy and safety of vaccines against SARS-CoV-2 variants B.1.351 (501Y.V2) in South Africa found that a two-dose regimen of the vaccine did not confer any protection against COVID-19 due to this viral variant, which may be due to residual neutralizing antibodies in affected populations [127]. These findings may be attributed to the immunoregulatory effects of CHIs against the SARS-CoV-2 vaccine response in the endemic area, mainly in South Africa [65].

There is argument regarding whether helminth coinfection leads to increased susceptibility and attenuated immunopathology of other pathogens exacerbated pathology due to higher infection burdens of SARS-CoV-2 infection [128]. Observational study showed that presence of helminth antigens in COVID-19 patients was associated with reduction of disease severity [128]. As well, helminth-induced increase of SARS-CoV-2-specific cytotoxic T cells activation in COVID-19 patients may facilitates the elimination of SARS-CoV-2-infected cells [128].

These outcomes indicated that CHIs can affect the immune response and disease severity in COVID-19 patients.

### 4.6. Helminth-Derived Molecules

Infection with parasitic helminths leads to potent activation and modulation of the host immune response [129]. This modulation of immunity by helminth infections may have bystander effects in altering, either suppressing or exacerbating, unrelated inflammatory processes. Various ongoing clinical trials are testing the therapeutic application of helminth infection in patients with inflammatory diseases, including inflammatory bowel disease and allergic disorders. Rather than the use of live helminth infection, with the potential for side effects, an alternative approach is to identify the immune modulatory molecules produced by helminths that can alter immune functions [129]. Helminth-derived molecules (HDMs) modulate helminth-host immune interactions by inhibiting the expression of TLR4 and release of pro-inflammatory cytokines [129]. HDMs suppress the signal transducer and activator of transcription 3 (STAT3) and NF-*κ*B signaling pathways, leading to a marked inhibition of inflammatory and pro-inflammatory release [130]. It has been shown that *F. hepatica* proteins downregulate NF-*κ*B pathway effectors and inflammatory cytokines while promoting survival in a mouse septic shock model [131]. As well, parasites inhibit TLR expression and interfering with both MyD88-dependent signaling and a pathway that ultimately diminishes NF-*κ*B activity. This downregulated NF-*κ*B activity impairs T-cell activation [132]. Thus, inhibition of a master regulator NF-*κ*B by HDMs could be a possible mechanism for the reduction of hyperinflammation in COVID-19 with CHIs.

The protein fraction of helminth secretory and excretory products and extracellular vesicles, such as the miRNA cargo protein, can inhibit the host metalloproteinase activity [101]. It has been reported that HDMs reduce viral pneumonia-induced lung inflammation [133]. Moreover, HDMs are also capable of preventing and attenuating metabolic disorders such as obesity, glucose intolerance, and IR through the activation of M2 macrophages [28].

SARS-CoV-2 binds to TLR4 as an entry-point for pneumocyte cells, with this binding being able to activate the expression of ACE2 [134]. Therefore, activation of TLR4 leads to more viral binding and entry through ACE2 in type II pneumocyte cells, causing a significant reduction in surfactant with the development of ARDS [135]. In addition, activated TLR4 induces hyperinflammation and CS through the MyD88-dependent pathway, leading to multi-organ damage [135]. Additionally, activation of platelet TLR4 by SARS-CoV-2 may lead to activation of pro-thrombotic cascades, and therefore TLR4 antagonists can attenuate COVID-19 complications [136], with prolonged TLR4 activations leading to a marked activation of STAT3 and NF-*κ*B signaling pathway and induction of pro-inflammatory activations [137]. A case–control study included 25 COVID-19 and 10 healthy controls illustrated that TLR4-dependent platelet activation contribute in the development of thrombosis and coagulopathy in severely affected COVID-19 patients compared to controls [138]. Therefore, inhibition of TLR4 by HDMs may reduce thrombotic events in COVID-19 patients.

Notoriously, bioinformatic analysis of genomic and transcriptome sequencing data sets identified putative genes encoding endocannabinoid biosynthetic and degradative enzymes in many parasitic nematodes [139]. Increased levels of endocannabinoids and the endocannabinoid-like molecule in infected lung and intestine. Inhibition of CB_1_R but not CB_2_R resulted in increased *Nippostrongylus brasiliensis* worm burden and egg output, associated with significantly decreased expression of the T helper type 2 cytokine IL-5 in intestinal tissue and splenocyte cultures [134]. Up to date, Al-kuraishy et al. [140] illustrated that cannabinoids might be used to manage COVID-19 because of their potent anti-inflammatory effects, suppressing pro-inflammatory cytokines and inhibiting inflammatory signaling pathways. Therefore, HDMs in COVID-19 patients with CHIs may be a protective against SARS-CoV-2 infection-induced hyperinflammation and immunoinflammatory disorders.

**Figure 3:**
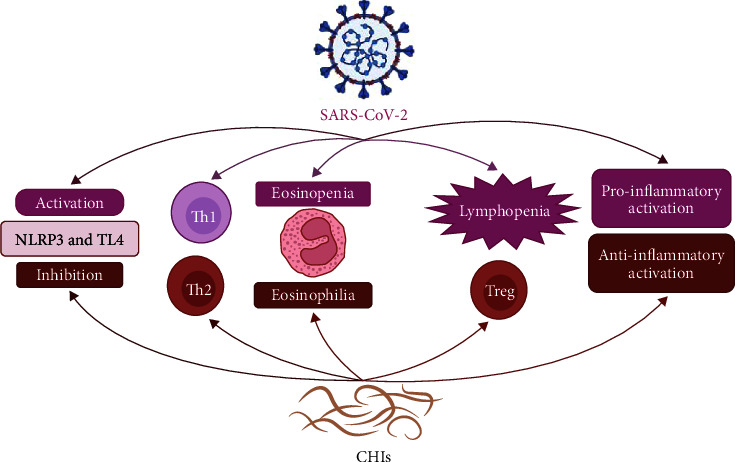
The interaction between COVID-19 and chronic helminth infections (CHIs): inhibitions of NLRP3 and TL4 activation with activation of type 2 immune response (Th2), anti-inflammatory cytokines, regulatory T cells (Treg) and eosinophilia by CHIs attenuate immunological disorders that are induced by COVID-19.

Thus, CHIs counteract COVID-19 severity along different lines, from local to systemic immune-modulating effects (Figure 3).

The present review had several limitations, including a paucity of clinical data, most of CHIs effect was speculative depending on preclinical data, and HDMs were not estimated from published researches and their correlation with COVID-19 severity. However, the strength of the present review gave a mechanistic role of CHIs on the SARS-CoV-2 infection and COVID-19 severity through modulation of immune response and damping of hyperinflammation.

## 5. Conclusion

Epidemiologically, helminths can multiply or reproduce within the human host and can survive for many years in tissues and intestinal niches. CHIs have systemic immunomodulatory effects triggered by their excretory/secretory helminth (E/S) products and may influence the severity of other infections. These helminth products (E/S) induce the host Th2 immune responsiveness mediated by Treg. The immunological tolerance to harmless and self-antigens is mediated by Treg, which are upregulated in CHIs. CHIs-mediated immune-modulating effects in COVID-19 including modulation of cytokine production toward anti-inflammatory axis with reduction of pro-inflammatory cytokines, downregulation of cell-surface interactions through activation of cytotoxic T cells and programed cell death, driving of human IgG to be noninflammatory IgG4 by Treg-dependent-Foxp3 production, activation of host cells to produce TGF*β*, which provokes Treg cell function; alteration in macrophage response from classically activated macrophage (M1) to alternatively ones (M2), where helminth-stimulated M2 leads to activation of Treg and induction of immune tolerance. Moreover, CHIs through HDMs may suppress SARS-CoV-2 entry and associated hyperinflammation through attenuation of the TLR4/NF-kB signaling pathway. CHIs may reduce the COVID-19 severity by reducing the SARS-CoV-2 entry points at ACE2/DPP4/CD147 axis in the initial phase and immunomodulation in the late phase of the disease by suppressingTLR4/NF-kB signaling pathway. Nevertheless, large-scale prospective studies are recommended to confirm the potential effect of CHIs on the pathological and clinical course of COVID-19.

## Data Availability

All data generated or analyzed during this study are included in this published article.
